# Dichloridoocta­kis(2-chloro­benz­yl)di-μ_2_-hydroxido-di-μ_3_-oxido-tetra­tin(IV)

**DOI:** 10.1107/S1600536809045176

**Published:** 2009-10-31

**Authors:** Qi-Jun Zhang, Han-Dong Yin, Li-Yuan Wen, Da-Qi Wang

**Affiliations:** a College of Chemistry and Chemical Engineering, Liaocheng University, Shandong 252059, People’s Republic of China

## Abstract

The title tetra­nuclear Sn^IV^ compound, [Sn_4_(C_7_H_6_Cl)_8_Cl_2_O_2_(OH)_2_], has site symmetry 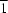. Two O^2−^ and two OH^−^ anions bridge four Sn^IV^ cations to form the tetra­nuclear compound. The two independent Sn^IV^ cations assume SnO_3_C_2_ and SnO_2_C_2_Cl distorted trigonal-bipyramidal coordination geometries. Intra­molecular O—H⋯Cl hydrogen bonding is present in the structure. One Cl atom of a chloro­benzyl ligand is disordered over two sites with an occupancy ratio of 0.693 (2):0.307 (2).

## Related literature

For a related structure, see: Li *et al.* (2006 ▶). For the corresponding bond distances in an organotin compound, see: Lo & Ng (2009 ▶).
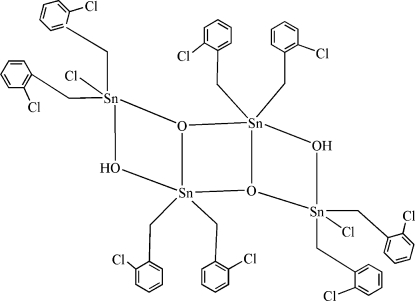


## Experimental

### 

#### Crystal data

[Sn_4_(C_7_H_6_Cl)_8_Cl_2_O_2_(OH)_2_]
*M* *_r_* = 1616.22
Triclinic, 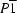

*a* = 10.986 (2) Å
*b* = 11.227 (2) Å
*c* = 13.573 (3) Å
α = 74.656 (2)°
β = 67.942 (2)°
γ = 75.753 (2)°
*V* = 1475.9 (6) Å^3^
*Z* = 1
Mo *K*α radiation
μ = 2.17 mm^−1^
*T* = 298 K
0.44 × 0.37 × 0.33 mm

#### Data collection

Bruker SMART CCD area-detector diffractometer
Absorption correction: multi-scan (*SADABS*; Sheldrick, 1996 ▶) *T*_min_ = 0.449, *T*_max_ = 0.535
7669 measured reflections
5112 independent reflections
3865 reflections with *I* > 2σ(*I*)
*R*_int_ = 0.015

#### Refinement

*R*[*F* ^2^ > 2σ(*F* ^2^)] = 0.029
*wR*(*F* ^2^) = 0.074
*S* = 1.04
5112 reflections
338 parameters
H-atom parameters constrained
Δρ_max_ = 1.27 e Å^−3^
Δρ_min_ = −0.57 e Å^−3^

### 

Data collection: *SMART* (Siemens, 1996 ▶); cell refinement: *SAINT* (Siemens, 1996 ▶); data reduction: *SAINT*; program(s) used to solve structure: *SHELXTL* (Sheldrick, 2008 ▶); program(s) used to refine structure: *SHELXTL*; molecular graphics: *SHELXTL*; software used to prepare material for publication: *SHELXTL*.

## Supplementary Material

Crystal structure: contains datablocks I, global. DOI: 10.1107/S1600536809045176/xu2652sup1.cif

Structure factors: contains datablocks I. DOI: 10.1107/S1600536809045176/xu2652Isup2.hkl

Additional supplementary materials:  crystallographic information; 3D view; checkCIF report

## Figures and Tables

**Table 1:** Selected bond lengths (Å)

Sn1—O1	2.148 (3)
Sn1—O2	2.050 (3)
Sn1—O2^i^	2.146 (3)
Sn1—C1	2.126 (5)
Sn1—C8	2.146 (5)
Sn2—O1	2.276 (3)
Sn2—O2	2.025 (3)
Sn2—C15	2.147 (5)
Sn2—C22	2.149 (5)
Sn2—Cl1	2.4376 (13)

Symmetry code: (i) 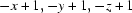.

**Table 2:** Hydrogen-bond geometry (Å, °)

*D*—H⋯*A*	*D*—H	H⋯*A*	*D*⋯*A*	*D*—H⋯*A*
O1—H1⋯Cl2	0.86	2.80	3.386 (4)	127

**Fig. 1.**
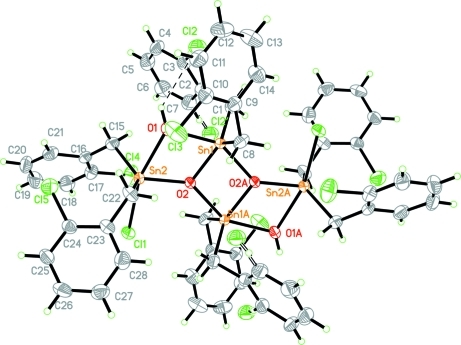
The molecular structure of the compound showing 50% probability displacement ellipsoids [symmetry code: (A) = 1-x, 1-y, 1-z].

## supplementary crystallographic information

### Comment

The title compound, (I), was obtained as an adventitious product of the partial hydrolysis of di(2-chlorobenzyl)dichloridotin(IV) during the attempted preparation of adducts of this tin precursor complex with 5-chlorosalicylaldehyde benzoyldrazone in benzene and ethanol. It crystallizes from dichloromethane and ethanol. From Fig. 1, it can be seen that complex (I) contains two independent penta-coordinated Sn atoms. It is a centrosymmetric complex, where one half of the molecule comprises the crystallographic asymmetric unit and the other half is generated by an inversion centre. Each of the two independent Sn atoms is five-coordinate, adopting approximate trigonal bipyramidal coordination (Table 1). These are similar to those in the related organotin compound (Li *et al.*, 2006). The molecular conformation is stabilized by O1—H1···Cl2 hydrogen bond (Table 2). The Sn—C distances lie in the rather narrow range 2.126 (5)–2.149 (5) Å, which are closed to the corresponding distances reported in the organotin compound (Lo & Ng, 2009).

### Experimental

Di(2-chlorobenzyl)dichloridotin(IV) (2 mmol) and 5-chlorosalicylaldehyde benzoyldrazone(2 mmol) was added to a solution of sodium methoxide (3 mmol) in benzene (15 ml) and ethanol (15 ml, 95%). The mixture was then heated under reflux with stirring for 5 h and the solvent was removed by evaporation in vacuo. The crude adduct was recrystallized from dichloromethane/ethanol and colourless crystals suitable for X-ray diffraction were obtained.

### Refinement

The H atoms were positioned geometrically, with methylene C—H distances of 0.97 Å, aromatic C—H distances of 0.93 Å, O—H distances of 0.862 Å and refined as riding on their parent atoms, with *U*_iso_(H) = 1.2 *U*_eq_(C,O). The Cl2 atom is disordered, the C2-phenyl part was refined as a rigid hexagon and the temperature factors of the carbon atoms were restrained to be nearly isotropic. The highest peak in the difference map is 1.21 Å apart from Cl1 atom.

### Crystal data

**Table:**

[Sn_4_(C_7_H_6_Cl)_8_Cl_2_O_2_(OH)_2_]	*Z* = 1
*M**_r_* = 1616.22	*F*(000) = 788
Triclinic, *P*1	*D*_x_ = 1.818 Mg m^−^^3^
Hall symbol: -P 1	Mo *K*α radiation, λ = 0.71073 Å
*a* = 10.986 (2) Å	Cell parameters from 4036 reflections
*b* = 11.227 (2) Å	θ = 2.6–27.4°
*c* = 13.573 (3) Å	µ = 2.17 mm^−^^1^
α = 74.656 (2)°	*T* = 298 K
β = 67.942 (2)°	Block, colourless
γ = 75.753 (2)°	0.44 × 0.37 × 0.33 mm
*V* = 1475.9 (6) Å^3^	

### Data collection

**Table:**

Bruker SMART CCD area-detector diffractometer	5112 independent reflections
Radiation source: fine-focus sealed tube	3865 reflections with *I* > 2σ(*I*)
graphite	*R*_int_ = 0.015
φ and ω scans	θ_max_ = 25.0°, θ_min_ = 1.7°
Absorption correction: multi-scan (*SADABS*; Sheldrick, 1996)	*h* = −13→13
*T*_min_ = 0.449, *T*_max_ = 0.535	*k* = −13→13
7669 measured reflections	*l* = −16→10

### Refinement

**Table:**

Refinement on *F*^2^	Primary atom site location: structure-invariant direct methods
Least-squares matrix: full	Secondary atom site location: difference Fourier map
*R*[*F*^2^ > 2σ(*F*^2^)] = 0.029	Hydrogen site location: inferred from neighbouring sites
*wR*(*F*^2^) = 0.074	H-atom parameters constrained
*S* = 1.04	*w* = 1/[σ^2^(*F*_o_^2^) + (0.0262*P*)^2^ + 2.2448*P*] where *P* = (*F*_o_^2^ + 2*F*_c_^2^)/3
5112 reflections	(Δ/σ)_max_ = 0.001
338 parameters	Δρ_max_ = 1.27 e Å^−^^3^
0 restraints	Δρ_min_ = −0.57 e Å^−^^3^

### Fractional atomic coordinates and isotropic or equivalent isotropic displacement parameters (Å^2^)

**Table:**

	*x*	*y*	*z*	*U*_iso_*/*U*_eq_	Occ. (<1)
Sn1	0.59257 (3)	0.37338 (3)	0.44839 (2)	0.03438 (10)	
Sn2	0.49384 (3)	0.58881 (3)	0.25330 (2)	0.03713 (10)	
Cl1	0.35586 (14)	0.79133 (11)	0.28147 (11)	0.0516 (3)	
Cl2	0.6318 (2)	0.0780 (2)	0.34868 (19)	0.0704 (6)	0.693 (2)
Cl2'	0.2326 (5)	0.3325 (5)	0.5648 (4)	0.0704 (6)	0.307 (2)
Cl3	0.96390 (18)	0.40745 (16)	0.17259 (15)	0.0897 (6)	
Cl4	0.09931 (19)	0.5877 (2)	0.36984 (14)	0.0927 (6)	
Cl5	0.6057 (2)	0.72167 (16)	−0.03870 (14)	0.0908 (6)	
O1	0.6121 (3)	0.3933 (3)	0.2815 (2)	0.0444 (8)	
H1	0.6408	0.3297	0.2503	0.053*	
O2	0.4776 (3)	0.5414 (3)	0.4119 (2)	0.0365 (7)	
C1	0.4860 (7)	0.2203 (6)	0.5202 (5)	0.077 (2)	
H1A	0.5486	0.1446	0.5314	0.092*	
H1B	0.4238	0.2336	0.5908	0.092*	
C2	0.4103 (7)	0.2003 (5)	0.4555 (5)	0.0597 (16)	
C3	0.4702 (6)	0.1302 (5)	0.3739 (5)	0.0646 (17)	
H3A	0.5605	0.0982	0.3588	0.077*	0.307 (2)
C4	0.4013 (7)	0.1078 (6)	0.3160 (5)	0.0697 (18)	
H4	0.4440	0.0592	0.2629	0.084*	
C5	0.2691 (8)	0.1581 (7)	0.3376 (6)	0.078 (2)	
H5	0.2210	0.1431	0.2998	0.093*	
C6	0.2087 (7)	0.2301 (6)	0.4146 (6)	0.0775 (19)	
H6	0.1197	0.2663	0.4277	0.093*	
C7	0.2776 (8)	0.2497 (6)	0.4731 (5)	0.0724 (18)	
H7A	0.2335	0.2980	0.5263	0.087*	0.693 (2)
C8	0.8018 (5)	0.3527 (5)	0.4200 (4)	0.0541 (14)	
H8A	0.8337	0.4296	0.3766	0.065*	
H8B	0.8166	0.3377	0.4887	0.065*	
C9	0.8788 (5)	0.2460 (5)	0.3625 (4)	0.0469 (12)	
C10	0.9489 (5)	0.2595 (5)	0.2531 (4)	0.0510 (13)	
C11	1.0141 (5)	0.1589 (6)	0.2019 (5)	0.0630 (16)	
H11	1.0598	0.1719	0.1278	0.076*	
C12	1.0104 (6)	0.0399 (6)	0.2620 (6)	0.080 (2)	
H12	1.0524	−0.0287	0.2285	0.096*	
C13	0.9448 (7)	0.0222 (6)	0.3713 (7)	0.086 (2)	
H13	0.9450	−0.0587	0.4121	0.103*	
C14	0.8791 (6)	0.1218 (6)	0.4213 (5)	0.0666 (16)	
H14	0.8337	0.1074	0.4955	0.080*	
C15	0.3720 (6)	0.5022 (5)	0.2102 (4)	0.0541 (14)	
H15A	0.4308	0.4523	0.1568	0.065*	
H15B	0.3287	0.4446	0.2740	0.065*	
C16	0.2673 (6)	0.5833 (5)	0.1664 (4)	0.0488 (13)	
C17	0.1427 (6)	0.6287 (5)	0.2294 (5)	0.0596 (15)	
C18	0.0470 (7)	0.7033 (6)	0.1868 (6)	0.0728 (18)	
H18	−0.0357	0.7343	0.2323	0.087*	
C19	0.0748 (7)	0.7312 (6)	0.0775 (6)	0.080 (2)	
H19	0.0110	0.7812	0.0480	0.096*	
C20	0.1997 (8)	0.6846 (6)	0.0096 (5)	0.0760 (19)	
H20	0.2185	0.7022	−0.0650	0.091*	
C21	0.2924 (6)	0.6141 (5)	0.0526 (5)	0.0632 (16)	
H21	0.3754	0.5846	0.0065	0.076*	
C22	0.6829 (5)	0.6492 (5)	0.1629 (4)	0.0546 (14)	
H22A	0.7318	0.6346	0.2126	0.066*	
H22B	0.7322	0.5957	0.1103	0.066*	
C23	0.6833 (5)	0.7819 (4)	0.1041 (4)	0.0445 (12)	
C24	0.6538 (5)	0.8249 (5)	0.0094 (4)	0.0491 (13)	
C25	0.6586 (6)	0.9448 (5)	−0.0481 (4)	0.0566 (14)	
H25	0.6375	0.9697	−0.1116	0.068*	
C26	0.6952 (6)	1.0277 (5)	−0.0102 (5)	0.0672 (17)	
H26	0.7000	1.1093	−0.0487	0.081*	
C27	0.7244 (7)	0.9914 (6)	0.0830 (5)	0.0712 (18)	
H27	0.7487	1.0480	0.1085	0.085*	
C28	0.7178 (6)	0.8692 (6)	0.1404 (5)	0.0666 (16)	
H28	0.7370	0.8454	0.2047	0.080*	

### Atomic displacement parameters (Å^2^)

**Table:**

	*U*^11^	*U*^22^	*U*^33^	*U*^12^	*U*^13^	*U*^23^
Sn1	0.0413 (2)	0.02862 (17)	0.03475 (19)	−0.00165 (14)	−0.01569 (15)	−0.00788 (13)
Sn2	0.0458 (2)	0.03431 (18)	0.03224 (19)	−0.00562 (15)	−0.01628 (15)	−0.00401 (14)
Cl1	0.0610 (8)	0.0372 (6)	0.0546 (8)	0.0030 (6)	−0.0240 (7)	−0.0085 (6)
Cl2	0.0727 (14)	0.0590 (12)	0.0858 (15)	−0.0032 (10)	−0.0315 (12)	−0.0237 (10)
Cl2'	0.0727 (14)	0.0590 (12)	0.0858 (15)	−0.0032 (10)	−0.0315 (12)	−0.0237 (10)
Cl3	0.0743 (11)	0.0692 (11)	0.0932 (13)	−0.0020 (9)	−0.0141 (10)	0.0080 (9)
Cl4	0.0917 (13)	0.1304 (16)	0.0571 (10)	−0.0280 (12)	−0.0216 (9)	−0.0155 (10)
Cl5	0.1487 (18)	0.0662 (10)	0.0763 (12)	−0.0408 (11)	−0.0494 (12)	−0.0049 (8)
O1	0.054 (2)	0.0381 (18)	0.0397 (19)	−0.0030 (16)	−0.0209 (16)	−0.0015 (14)
O2	0.0440 (19)	0.0304 (16)	0.0341 (17)	0.0006 (14)	−0.0169 (15)	−0.0051 (13)
C1	0.137 (6)	0.064 (4)	0.056 (4)	−0.059 (4)	−0.052 (4)	0.014 (3)
C2	0.097 (5)	0.048 (3)	0.048 (3)	−0.043 (3)	−0.034 (3)	0.011 (3)
C3	0.081 (5)	0.060 (4)	0.067 (4)	−0.028 (3)	−0.041 (4)	0.003 (3)
C4	0.099 (5)	0.068 (4)	0.063 (4)	−0.032 (4)	−0.038 (4)	−0.013 (3)
C5	0.093 (6)	0.082 (5)	0.084 (5)	−0.041 (4)	−0.051 (4)	−0.002 (4)
C6	0.074 (5)	0.074 (4)	0.090 (5)	−0.029 (4)	−0.031 (4)	−0.003 (4)
C7	0.101 (6)	0.065 (4)	0.061 (4)	−0.042 (4)	−0.027 (4)	0.000 (3)
C8	0.047 (3)	0.063 (4)	0.064 (4)	0.000 (3)	−0.022 (3)	−0.033 (3)
C9	0.035 (3)	0.054 (3)	0.055 (3)	−0.001 (2)	−0.019 (2)	−0.016 (3)
C10	0.039 (3)	0.058 (3)	0.058 (4)	−0.003 (3)	−0.020 (3)	−0.013 (3)
C11	0.045 (3)	0.075 (4)	0.068 (4)	0.005 (3)	−0.015 (3)	−0.031 (3)
C12	0.062 (4)	0.068 (4)	0.108 (6)	0.004 (3)	−0.016 (4)	−0.044 (4)
C13	0.069 (5)	0.052 (4)	0.112 (6)	−0.008 (3)	−0.016 (4)	0.003 (4)
C14	0.051 (4)	0.064 (4)	0.069 (4)	0.004 (3)	−0.014 (3)	−0.007 (3)
C15	0.065 (4)	0.048 (3)	0.060 (4)	−0.013 (3)	−0.029 (3)	−0.013 (3)
C16	0.061 (4)	0.045 (3)	0.055 (3)	−0.013 (3)	−0.030 (3)	−0.014 (2)
C17	0.063 (4)	0.067 (4)	0.057 (4)	−0.013 (3)	−0.026 (3)	−0.015 (3)
C18	0.064 (4)	0.079 (4)	0.085 (5)	−0.005 (3)	−0.038 (4)	−0.020 (4)
C19	0.082 (5)	0.077 (5)	0.095 (6)	−0.004 (4)	−0.057 (5)	−0.005 (4)
C20	0.106 (6)	0.083 (5)	0.058 (4)	−0.030 (4)	−0.045 (4)	−0.005 (3)
C21	0.066 (4)	0.063 (4)	0.074 (4)	−0.011 (3)	−0.030 (3)	−0.025 (3)
C22	0.047 (3)	0.046 (3)	0.058 (3)	−0.004 (2)	−0.016 (3)	0.005 (3)
C23	0.033 (3)	0.041 (3)	0.048 (3)	−0.006 (2)	−0.006 (2)	−0.002 (2)
C24	0.049 (3)	0.044 (3)	0.047 (3)	−0.008 (2)	−0.009 (3)	−0.006 (2)
C25	0.064 (4)	0.046 (3)	0.048 (3)	−0.005 (3)	−0.013 (3)	−0.002 (3)
C26	0.072 (4)	0.039 (3)	0.070 (4)	−0.002 (3)	−0.007 (3)	−0.007 (3)
C27	0.089 (5)	0.065 (4)	0.062 (4)	−0.033 (4)	−0.011 (4)	−0.016 (3)
C28	0.065 (4)	0.076 (4)	0.061 (4)	−0.020 (3)	−0.024 (3)	−0.005 (3)

### Geometric parameters (Å, °)

**Table:**

Sn1—O1	2.148 (3)	C9—C14	1.412 (7)
Sn1—O2	2.050 (3)	C10—C11	1.385 (8)
Sn1—O2^i^	2.146 (3)	C11—C12	1.370 (8)
Sn1—C1	2.126 (5)	C11—H11	0.9300
Sn1—C8	2.146 (5)	C12—C13	1.368 (9)
Sn2—O1	2.276 (3)	C12—H12	0.9300
Sn2—O2	2.025 (3)	C13—C14	1.367 (9)
Sn2—C15	2.147 (5)	C13—H13	0.9300
Sn2—C22	2.149 (5)	C14—H14	0.9300
Sn2—Cl1	2.4376 (13)	C15—C16	1.489 (7)
Cl2—C3	1.658 (7)	C15—H15A	0.9700
Cl2—H3A	0.7301	C15—H15B	0.9700
Cl2'—C7	1.606 (8)	C16—C17	1.368 (8)
Cl2'—H7A	0.7242	C16—C21	1.424 (7)
Cl3—C10	1.737 (6)	C17—C18	1.381 (8)
Cl4—C17	1.742 (6)	C18—C19	1.361 (9)
Cl5—C24	1.735 (5)	C18—H18	0.9300
O1—H1	0.8590	C19—C20	1.399 (9)
C1—C2	1.503 (7)	C19—H19	0.9300
C1—H1A	0.9700	C20—C21	1.345 (8)
C1—H1B	0.9700	C20—H20	0.9300
C2—C7	1.379 (9)	C21—H21	0.9300
C2—C3	1.391 (8)	C22—C23	1.492 (6)
C3—C4	1.379 (7)	C22—H22A	0.9700
C3—H3A	0.9301	C22—H22B	0.9700
C4—C5	1.371 (9)	C23—C24	1.377 (7)
C4—H4	0.9300	C23—C28	1.388 (7)
C5—C6	1.359 (9)	C24—C25	1.369 (7)
C5—H5	0.9300	C25—C26	1.374 (8)
C6—C7	1.371 (8)	C25—H25	0.9300
C6—H6	0.9300	C26—C27	1.355 (8)
C7—H7A	0.9300	C26—H26	0.9300
C8—C9	1.502 (7)	C27—C28	1.391 (8)
C8—H8A	0.9700	C27—H27	0.9300
C8—H8B	0.9700	C28—H28	0.9300
C9—C10	1.379 (7)		
			
O2—Sn1—C1	114.2 (2)	C10—C9—C8	124.6 (5)
O2—Sn1—O2^i^	73.49 (12)	C14—C9—C8	119.6 (5)
C1—Sn1—O2^i^	97.25 (19)	C9—C10—C11	123.1 (5)
O2—Sn1—C8	123.37 (17)	C9—C10—Cl3	120.6 (4)
C1—Sn1—C8	122.4 (3)	C11—C10—Cl3	116.3 (5)
O2^i^—Sn1—C8	97.54 (16)	C12—C11—C10	119.1 (6)
O2—Sn1—O1	74.51 (12)	C12—C11—H11	120.5
C1—Sn1—O1	100.54 (17)	C10—C11—H11	120.5
O2^i^—Sn1—O1	147.55 (11)	C13—C12—C11	119.8 (6)
C8—Sn1—O1	95.50 (17)	C13—C12—H12	120.1
O2—Sn2—C15	114.44 (17)	C11—C12—H12	120.1
O2—Sn2—C22	108.14 (17)	C14—C13—C12	120.9 (6)
C15—Sn2—C22	131.7 (2)	C14—C13—H13	119.6
O2—Sn2—O1	72.21 (11)	C12—C13—H13	119.6
C15—Sn2—O1	85.91 (16)	C13—C14—C9	121.3 (6)
C22—Sn2—O1	86.30 (16)	C13—C14—H14	119.4
O2—Sn2—Cl1	90.33 (9)	C9—C14—H14	119.4
C15—Sn2—Cl1	102.16 (15)	C16—C15—Sn2	118.8 (3)
C22—Sn2—Cl1	99.22 (15)	C16—C15—H15A	107.6
O1—Sn2—Cl1	162.53 (9)	Sn2—C15—H15A	107.6
C3—Cl2—H3A	3.0	C16—C15—H15B	107.6
C7—Cl2'—H7A	15.7	Sn2—C15—H15B	107.6
Sn1—O1—Sn2	100.13 (13)	H15A—C15—H15B	107.0
Sn1—O1—H1	120.9	C17—C16—C21	115.9 (5)
Sn2—O1—H1	137.0	C17—C16—C15	124.1 (5)
Sn2—O2—Sn1	112.76 (14)	C21—C16—C15	119.9 (5)
Sn2—O2—Sn1^i^	139.33 (14)	C16—C17—C18	123.0 (6)
Sn1—O2—Sn1^i^	106.51 (12)	C16—C17—Cl4	119.2 (4)
C2—C1—Sn1	114.9 (3)	C18—C17—Cl4	117.8 (5)
C2—C1—H1A	108.5	C19—C18—C17	119.4 (6)
Sn1—C1—H1A	108.5	C19—C18—H18	120.3
C2—C1—H1B	108.5	C17—C18—H18	120.3
Sn1—C1—H1B	108.5	C18—C19—C20	119.8 (6)
H1A—C1—H1B	107.5	C18—C19—H19	120.1
C7—C2—C3	115.8 (5)	C20—C19—H19	120.1
C7—C2—C1	122.3 (6)	C21—C20—C19	119.8 (6)
C3—C2—C1	121.9 (6)	C21—C20—H20	120.1
C4—C3—C2	122.6 (6)	C19—C20—H20	120.1
C4—C3—Cl2	121.9 (6)	C20—C21—C16	122.0 (6)
C2—C3—Cl2	115.4 (5)	C20—C21—H21	119.0
C4—C3—H3A	119.9	C16—C21—H21	119.0
C2—C3—H3A	117.5	C23—C22—Sn2	118.1 (3)
Cl2—C3—H3A	2.4	C23—C22—H22A	107.8
C5—C4—C3	119.1 (6)	Sn2—C22—H22A	107.8
C5—C4—H4	120.5	C23—C22—H22B	107.8
C3—C4—H4	120.5	Sn2—C22—H22B	107.8
C6—C5—C4	119.7 (6)	H22A—C22—H22B	107.1
C6—C5—H5	120.2	C24—C23—C28	115.7 (5)
C4—C5—H5	120.2	C24—C23—C22	122.7 (5)
C5—C6—C7	120.7 (7)	C28—C23—C22	121.5 (5)
C5—C6—H6	119.7	C25—C24—C23	123.7 (5)
C7—C6—H6	119.7	C25—C24—Cl5	118.1 (4)
C6—C7—C2	122.1 (7)	C23—C24—Cl5	118.1 (4)
C6—C7—Cl2'	130.8 (7)	C24—C25—C26	118.6 (5)
C2—C7—Cl2'	107.1 (5)	C24—C25—H25	120.7
C6—C7—H7A	119.0	C26—C25—H25	120.7
C2—C7—H7A	119.0	C27—C26—C25	120.4 (5)
Cl2'—C7—H7A	12.1	C27—C26—H26	119.8
C9—C8—Sn1	111.3 (3)	C25—C26—H26	119.8
C9—C8—H8A	109.4	C26—C27—C28	119.8 (6)
Sn1—C8—H8A	109.4	C26—C27—H27	120.1
C9—C8—H8B	109.4	C28—C27—H27	120.1
Sn1—C8—H8B	109.4	C23—C28—C27	121.7 (6)
H8A—C8—H8B	108.0	C23—C28—H28	119.2
C10—C9—C14	115.9 (5)	C27—C28—H28	119.2
			
O2—Sn1—O1—Sn2	4.51 (11)	Sn1—C8—C9—C10	100.7 (5)
C1—Sn1—O1—Sn2	116.9 (2)	Sn1—C8—C9—C14	−77.6 (5)
O2^i^—Sn1—O1—Sn2	−5.2 (3)	C14—C9—C10—C11	1.2 (8)
C8—Sn1—O1—Sn2	−118.63 (17)	C8—C9—C10—C11	−177.1 (5)
O2—Sn2—O1—Sn1	−4.61 (11)	C14—C9—C10—Cl3	−176.6 (4)
C15—Sn2—O1—Sn1	−121.91 (19)	C8—C9—C10—Cl3	5.0 (7)
C22—Sn2—O1—Sn1	105.78 (19)	C9—C10—C11—C12	−0.5 (9)
Cl1—Sn2—O1—Sn1	−3.5 (4)	Cl3—C10—C11—C12	177.4 (5)
C15—Sn2—O2—Sn1	82.0 (2)	C10—C11—C12—C13	−1.2 (10)
C22—Sn2—O2—Sn1	−74.7 (2)	C11—C12—C13—C14	2.0 (11)
O1—Sn2—O2—Sn1	5.17 (13)	C12—C13—C14—C9	−1.3 (10)
Cl1—Sn2—O2—Sn1	−174.49 (13)	C10—C9—C14—C13	−0.3 (8)
C15—Sn2—O2—Sn1^i^	−114.1 (3)	C8—C9—C14—C13	178.1 (6)
C22—Sn2—O2—Sn1^i^	89.3 (3)	O2—Sn2—C15—C16	118.1 (4)
O1—Sn2—O2—Sn1^i^	169.1 (3)	C22—Sn2—C15—C16	−92.2 (5)
Cl1—Sn2—O2—Sn1^i^	−10.5 (2)	O1—Sn2—C15—C16	−173.6 (4)
C1—Sn1—O2—Sn2	−100.2 (2)	Cl1—Sn2—C15—C16	22.1 (5)
O2^i^—Sn1—O2—Sn2	169.2 (2)	Sn2—C15—C16—C17	−83.9 (6)
C8—Sn1—O2—Sn2	81.0 (2)	Sn2—C15—C16—C21	98.5 (5)
O1—Sn1—O2—Sn2	−5.41 (13)	C21—C16—C17—C18	−2.0 (8)
C1—Sn1—O2—Sn1^i^	90.6 (2)	C15—C16—C17—C18	−179.7 (5)
O2^i^—Sn1—O2—Sn1^i^	0.000 (1)	C21—C16—C17—Cl4	176.0 (4)
C8—Sn1—O2—Sn1^i^	−88.2 (2)	C15—C16—C17—Cl4	−1.6 (7)
O1—Sn1—O2—Sn1^i^	−174.57 (16)	C16—C17—C18—C19	1.9 (9)
O2—Sn1—C1—C2	55.6 (6)	Cl4—C17—C18—C19	−176.2 (5)
O2^i^—Sn1—C1—C2	130.8 (5)	C17—C18—C19—C20	−0.2 (10)
C8—Sn1—C1—C2	−125.5 (5)	C18—C19—C20—C21	−1.1 (10)
O1—Sn1—C1—C2	−22.0 (6)	C19—C20—C21—C16	0.9 (9)
Sn1—C1—C2—C7	−94.6 (6)	C17—C16—C21—C20	0.6 (8)
Sn1—C1—C2—C3	85.5 (6)	C15—C16—C21—C20	178.4 (5)
C7—C2—C3—C4	−1.9 (8)	O2—Sn2—C22—C23	−116.2 (4)
C1—C2—C3—C4	178.0 (5)	C15—Sn2—C22—C23	92.7 (5)
C7—C2—C3—Cl2	176.7 (4)	O1—Sn2—C22—C23	173.9 (4)
C1—C2—C3—Cl2	−3.4 (7)	Cl1—Sn2—C22—C23	−22.8 (4)
C2—C3—C4—C5	1.3 (9)	Sn2—C22—C23—C24	−74.9 (6)
Cl2—C3—C4—C5	−177.2 (5)	Sn2—C22—C23—C28	107.0 (5)
C3—C4—C5—C6	0.7 (9)	C28—C23—C24—C25	0.8 (8)
C4—C5—C6—C7	−2.0 (10)	C22—C23—C24—C25	−177.4 (5)
C5—C6—C7—C2	1.3 (9)	C28—C23—C24—Cl5	−178.4 (4)
C5—C6—C7—Cl2'	177.9 (6)	C22—C23—C24—Cl5	3.5 (7)
C3—C2—C7—C6	0.6 (8)	C23—C24—C25—C26	0.2 (9)
C1—C2—C7—C6	−179.3 (5)	Cl5—C24—C25—C26	179.3 (4)
C3—C2—C7—Cl2'	−176.7 (4)	C24—C25—C26—C27	−0.8 (9)
C1—C2—C7—Cl2'	3.3 (7)	C25—C26—C27—C28	0.3 (10)
O2—Sn1—C8—C9	−131.3 (3)	C24—C23—C28—C27	−1.3 (8)
C1—Sn1—C8—C9	50.0 (5)	C22—C23—C28—C27	176.9 (5)
O2^i^—Sn1—C8—C9	153.6 (4)	C26—C27—C28—C23	0.7 (10)
O1—Sn1—C8—C9	−56.2 (4)		

Symmetry codes: (i) −*x*+1, −*y*+1, −*z*+1.

### Hydrogen-bond geometry (Å, °)

**Table:**

*D*—H···*A*	*D*—H	H···*A*	*D*···*A*	*D*—H···*A*
O1—H1···Cl2	0.86	2.80	3.386 (4)	127
